# New insight into the role of lipid metabolism-related proteins in rheumatic heart valve disease

**DOI:** 10.1186/s12944-022-01722-x

**Published:** 2022-10-28

**Authors:** Ting Xie, Xuan Chen, Cong Liu, Xingjiu Cai, Mei Xiang, Shiwu Liu, Ruzheng Li, Zhichuan Lin, Debing Liu, Ming Dong, Xinzhong Chen, Minghui Zou, Ping Qiao

**Affiliations:** 1 Department of Cardiac Surgery, Hainan General Hospital, Hainan Affiliated Hospital of Hainan Medical University, No.19 Xiuhua Road, Xiuying District, Haikou, 571000 Hainan China; 2 International College of Nursing, Hainan Vocational University of Science and Technology, Haikou, Hainan China; 3 Department of Ultrasound Medicine, Union Hospital, Tongji Medical College, Huazhong University of Science and Technology, Wuhan, Hubei China; 4 Department of Cardiovascular Medicine, Hainan General Hospital, No.19 Xiuhua Road, Xiuying District, Haikou, 571000 Hainan China; 5 Department of Neurology, Hainan General Hospital, Haikou, Hainan China; 6 Department of Cardiovascular Surgery, Union Hospital, Tongji Medical College, Huazhong University of Science and Technology, Wuhan, Hubei China; 7 Department of Cardiovascular Surgery, Guangzhou Women & Children’s Medical Center, Guangzhou, Guangdong China

**Keywords:** Rheumatic mitral valve disease, Lipid metabolism, Immunohistochemical staining, Hematoxylin–eosin staining

## Abstract

**Purpose:**

The aim of this study was to determine the expression of lipid metabolism-related proteins in rheumatic heart valve disease (RHVD).

**Methods:**

This retrospective study involved a total of 20 cases of moderate or severe rheumatic mitral valve stenosis and 4 cases of mitral regurgitation due to secondary causes from September 2018 to September 2021. The patients enrolled included 12 males and 12 females who underwent surgical excision of the mitral valve at the cardiac surgery department of Hainan General Hospital. The samples of mitral valve were collected during surgery treatment as the study group, and mitral valves collected from patients with ischemic heart disease were allocated into the control group. Hematoxylin–eosin (HE), oil red staining and immunohistochemical (IHC) staining were conducted to compare the expression of lipid metabolism-related proteins (ATP-binding cassette transporter A1 and acyl-coenzyme A: cholesterol acyltransferase-1), and real-time polymerase chain reaction (RT–PCR) was applied to compare the mRNA levels of ABCA1, ACAT1, and the inflammatory cytokines TNF-α, IL-10, and MCP-1.

**Results:**

In general, the rheumatic mitral valve showed leaflet thickening along with border adhesions and visible yellow fats. Oil red O staining also revealed the abovementioned results as well as fat cells. Both ABCA1 and ACAT1 were expressed in the rheumatic mitral valve via IHC, whereas only ACAT1 showed a faint level of expression in the ischemic mitral valve with no expression of ABCA1. In addition, compared with the ischemic mitral valve, RT-PCT showed increased mRNA expression levels of ABCA1, ACAT1, and the inflammatory cytokines TNF-α, IL-10, and MCP-1 (*P* < 0.05). After dividing the RMs into two groups for RT–PCR, we found that the higher the expression of ABCA1 and ACAT1 was, the lower the relative expression of inflammatory factors.

**Conclusion:**

This study showed that adipose tissue, adipose cells, and lipid transport-related proteins were expressed strongly in the rheumatic mitral valve, suggesting that adipose tissue formation might be one of the important pathways in the pathology of rheumatic heart disease. In addition, adipose tissue and adipocytes were also involved in the inflammatory process. These data provide new insight into pathological mechanisms in rheumatic heart disease.

## Introduction

Rheumatic valvular heart disease (RHVD) is acknowledged as a postinfectious sequel of acute rheumatic fever following the abnormal immune response to streptococcal pharyngitis that triggers valvular damage. Previous studies are available to show that RHVD is still the leading cause of cardiovascular death among children and young adults in low- and middle-income countries [1, 2]. RHVD is diagnosed thorough clinical investigation and echocardiographic results and is primarily treated by medicine; in severe cases, interventional techniques and surgical treatment might be needed [3]. However, there is currently no cure for rheumatic heart disease, and damage to the heart valves often lasts a lifetime.

Studies have shown that lipid metabolism is closely related to the inflammatory process of rheumatic diseases and autoimmune reactions such as gout and autoimmune rheumatic disease (ARD) [4], but the potential role of metabolism in rheumatic heart valves has not been elucidated. ATP-binding cassette transporter A1 (ABCA1), which mediates the efflux of cellular cholesterol and phospholipids, is the rate-limiting step in lipid metabolism. Acyl-coenzyme A:cholesterol acyltransferase-1 (ACAT1) is able to promote the accumulation of cholesterol ester in macrophages, thereby resulting in foam cell formation [5]. Adipose tissue can not only store energy but is also involved in regulating immunity and inflammation, which is usually insufficient in normal heart valves. However, pale yellow adipose tissue was found in damaged rheumatic heart valves, partially suggesting that fat may contribute to the development of rheumatic heart valve diseases. One of earlier studies on the lipid metabolism mechanism of RHVD showed that drugs or dietary management could prevent or treat RHVD effectively [6]. As ACAT1 and ABCA1 are important cholesterol transporters in lipid metabolism [5], it is important to determine their potential association with the condition.

In this study, we aimed to detect the expression of the lipid metabolism-related proteins ACAT1 and ABCA1 to provide evidence for the role of lipid metabolism in RHD. Our study provides new insight into the underlying mechanisms and forms a basis for new therapeutic regimens for the treatment of RHVD in the future.

## Methods and materials

### Patients

In total, there were 20 cases of moderate and severe rheumatic mitral valve stenosis and 4 cases of mitral regurgitation due to secondary causes from September 2018 to September 2021 in this retrospective study. The mitral valve collected from patients with ischemic heart disease was selected as the control group. Inclusion criteria: patients with a history of rheumatoid arthritis, palpitations, shortness of breath and other symptoms before admission; patients with mitral valve stenosis and thickened valve leaflets by echocardiography. Exclusion criteria: patients with rheumatic activity were excluded, including patients with anti-0 > 116 IU/L or patients with joint redness, swelling, heat and pain.

All patients underwent surgical excision of the mitral valve at the cardiac department of Hainan Provincial Hospital. All tissue specimens were collected with the informed consent of the patients and family members. The study protocol was performed in accordance with the Declaration of Helsinki and approved by the Ethics Committee of Hainan General Hospital. Informed consent was obtained from all the study subjects before enrollment.

#### Inclusion criteria

Enrolled patients were diagnosed with rheumatic heart disease [7] that invaded the valvula bicuspidalis and caused mitral valve stenosis.

#### Exclusion criteria

(1) traumatic mitral valve, mitral valve degeneration, infective endocarditis, and other kinds of damaged mitral valve; (2) patient with parathyroid diseases, renal failure and other systemic diseases affecting the transport of calcium and phosphorus.

## Methods

### Sample collection

Samples were collected during surgery. All samples in the different groups were divided into two parts. One cusp was fixed in 10% formaldehyde for histological processing, whereas the other was frozen in liquid nitrogen. Tissues fixed in formaldehyde were embedded in paraffin. Valve samples were then excised vertically from the base to the midpoint of the leaflet. Five-micrometer-thick sections were obtained and stained with hematoxylin–eosin (H&E) or immunohistochemical (IHC). All sections were evaluated by two or more qualified pathologists and finally reviewed by the chief physician.

### HE staining and general specimen observation

With conventional HE staining, the thickness of the valve was observed under an optical microscope. Tissue structure, cell composition, calcification site and changes around the calcification were observed and assessed by the naked eye.

### Oil red O staining

The samples underwent the following process by oil red staining: (1) frozen sections; (2) washing with distilled water; (3) immerging in oil red diluent for 10-15 minutes, avoiding light, sealed; (4) differentiation to clear stroma in 60% ethanol; (5) washing; (6) Marry’s hematoxylin restained nuclei; (8) washing with water; and (9) sealing with glycerin or glycerin gelatin.

### Immunohistochemical staining

Rabbit anti-human ACAT1 and ABCA1 antibodies were purchased from Beijing Booshen Biotechnology Co., Ltd., and the antibodies were diluted 1:100 using a PV-9000 universal two-step kit. Slides were then incubated with a biotin-conjugated anti-mouse or anti-rabbit immunoglobulin antibody, followed by HRP-conjugated streptavidin and ABC substrate.

### RT–PCR detection

In cryostat sections, RNA was extracted and quantified by real-time polymerase chain reaction (RT–PCR) analysis.

Total RNA was extracted routinely, and 1 μg total RNA per 20 UL system was used as a template for reverse transcription to synthesize cRNA with the following conditions: 25 °C for 10 min; 37 °C for 120 min; 85 °C for 5 s; and − 80 °C. Real-time quantitative PCR was performed with 2 μL reverse transcription products. The reaction system was 25 μL with GAPDH as an internal reference. Briefly, two-step amplification conditions were used as follows: predenaturation at 95 °C for 30 seconds and 10 seconds, followed by amplification at 60 °C for 30 seconds, for a total of 40 cycles. (See Table 1 for primers).

**Table 1:** The primers of RT-PCR

Accession No.	Gene	Primer sequence (5′ to 3′)		Annealing temperature (°C)
NM_002046	H-GAPDH-S	GGAAGCTTGTCATCAATGGAAATC	168	60
	H-GAPDH-A	TGATGACCCTTTTGGCTCCC		60
NM_001251.2	H-CD68(rz)-S	CCAACAAAACCAAGGTCCAGG	99	60
	H-CD68(rz)-A	GGTCCTGCATGAATCCAAAGC		60
NM_000019.4	H-ACAT1-S	AAGTAAAGCAGCATGGGAAGC	267	60
	H-ACAT1-A	GCCAGTGGTGTAACATTGAGCC		60
XM_011518342.3	H-ABCA1-S	ATGGCTGATGCCCTGGAAAG	117	60
	H-ABCA1-A	AATGAGGAAGAACACCACCCC		60
NM_000594.3	H-TNF-α-S	TCTACTCCCAGGTCCTCTTCAAG	201	60
	H-TNF-α-A	GGAAGACCCCTCCCAGATAGA		60
NM_000572.3	H-Il-10(RZ)-S	ACTGCTCTGTTGCCTGGTCCT	232	60
	H-Il-10(RZ)-A	GGCTTGGCAACCCAGGTAA		60
NM_002982.3	H-MCP-1-S	CTCGCTCAGCCAGATGCAAT	113	60
	H-MCP-1-A	CACTTGCTGCTGGTGATTCTTCT		60

*GAPDH* glyceraldehyde-3-phosphate dehydrogenase, *ACAT1* acetoacetyl Coenzyme A thiolase −1, *ABCA1* ATP binding cassette subfamily A member 1, *TNF-α* tumour necrosis factor α, *IL-10* interleukin10, *MCP-1* Monocyte chemoattractant protein-1

### Statistical analyses

Statistical analysis was performed using SPSS software. Continuous data are presented as the mean ± standard deviation in the normal distribution and as the median and interquartile range for the nonnormal distribution. The categorical data are presented as prevalence (%). The Kolmogorov–Smirnov test was performed to assess conformity with a normal distribution. Unpaired categorical variables were compared by Fisher’s exact test. The t test or Mann–Whitney test was applied to determine differences between continuous variables. *P* < 0.05 was considered statistically significant.

## Results

### Clinical information

A total of 20 cases of moderate to severe rheumatic mitral stenosis and 4 cases of secondary mitral regurgitation between September 2018 and September 2021 were included. The basic clinical data of the patients were collected, and Fisher’s exact test by statistical analysis showed that compared with the control group, the study group had no significant difference in sex, smoking, alcohol use, diabetes, hyperlipidemia or hypertension (*P* > 0.05) (Table 2).

**Table 2:** The demographical and clinical parameters of the patients

Index	Study group (20)	Control group (4)	X^2^	*P*
F/M	10/10	2/2	0	1
Smoking	2	1	0.686	0.437
Alcohol use	3	1	0.24	0.544
Diabetes	2	1	0.686	0.437
Hypertension	3	2	2.476	0.179
Hyperlipidemia	2	1	0.686	0.437

### HE staining

**Fig. 1:**
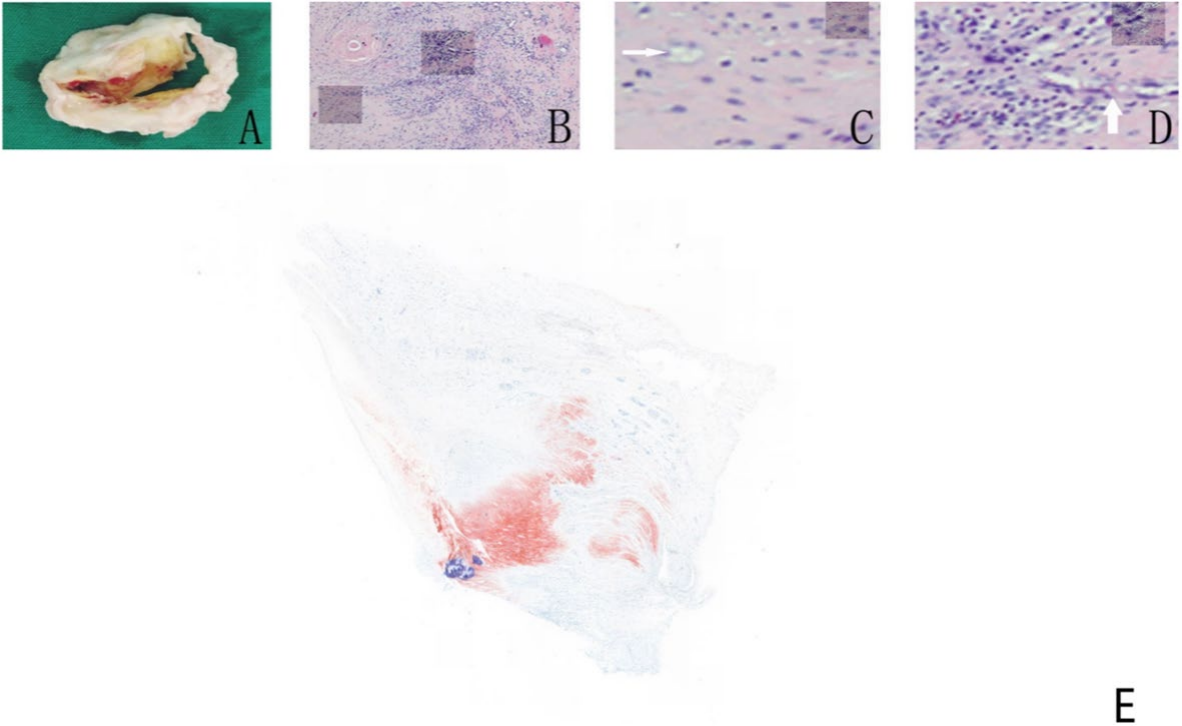
General specimen and HE/oil red staining. **A** Rheumatic mitral valve shows thickening, adhesions, visible yellow fat and calcification; **B** HE staining (× 40). Rheumatic valves show peri-fibrous tissue arranged disorderly; **C**, fat-like cells (× 100); **D** neovascularization. (× 100); **E** rheumatic mitral valve oil red staining

On gross observation, ischemic mitral valves appeared thin and transparent. The rheumatic mitral valve showed leaflet thickening along with border adhesions, visible yellow fats and calcification (Fig. 1A). HE staining of normal valves showed avascular and neatly arranged fibers with no inflammatory cell infiltration. The rheumatic mitral valve presented a large amount of inflammatory cell infiltration with visible rheumatoid nodules and fibrous tissue disordering the original structure of the valve (Fig. 1B). Further observation revealed adipocytes (Fig. 1C) and neovascularization in the damaged valves (Fig. 1D).

### Oil red O staining

Oil red O staining showed that in the area of rheumatic mitral valve injury, the fat was bright red. In the control group, no fat was found (Fig. 1E).

### Immunohistochemical analysis

**Fig. 2:**
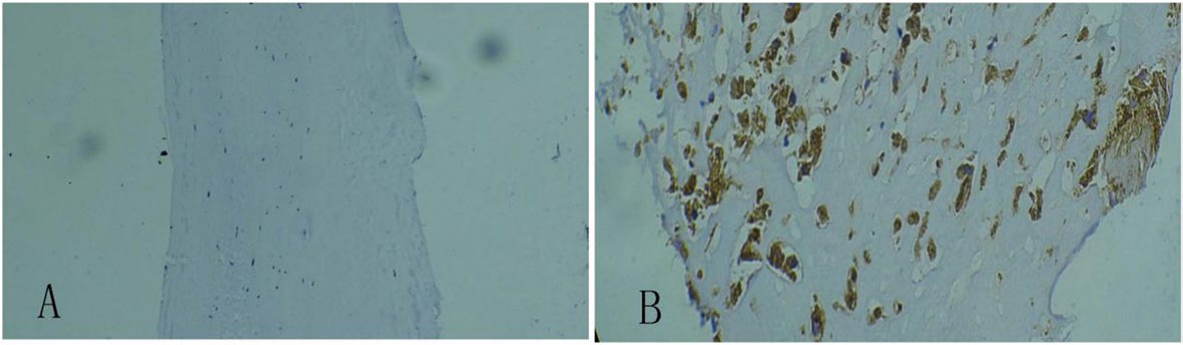
Immunohistochemical staining of CD68. The expression of CD68 was not found in ischemic mitral valve (**A**, × 40) but was found in rheumatic mitral valve n. (**B**, × 100)

**Fig. 3:**
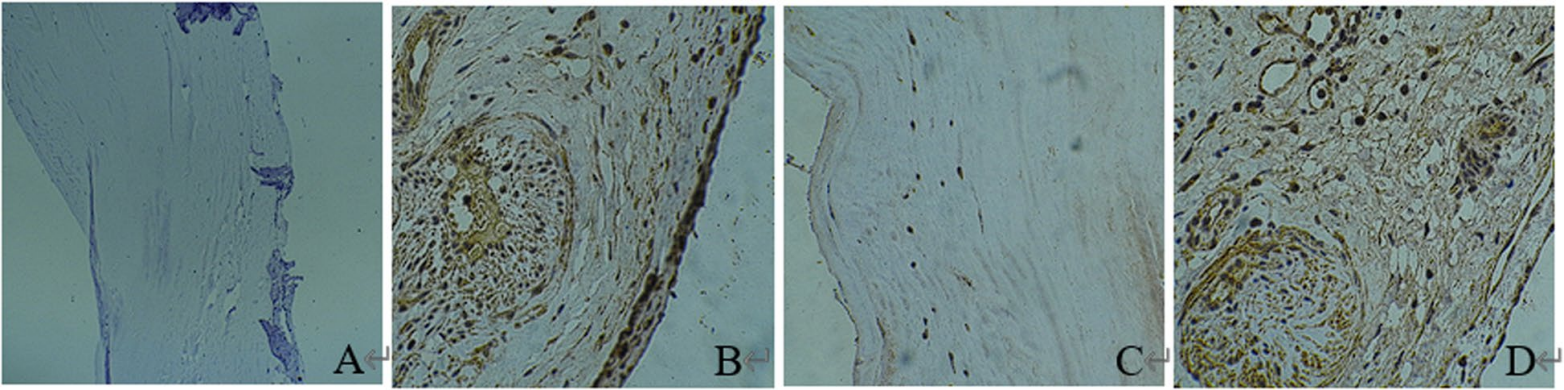
Immunohistochemical staining of ABCA1 and ACAC1. The expression of ABCA1 was not found in the ischemic mitral valve (**A**, × 40). ABCA1 expression and rheumatoid nodules in the rheumatic mitral valve (**B**, × 40). The expression of ACAT1 was weakly positive in the ischemic mitral valve (**C** × 40. Increased expression of ACAT1 was found in the rheumatic mitral valve (**D**, × 40)

In the rheumatic mitral valve, CD68 is a well-known macrophage marker expressed in the cytoplasm (Fig. 2A). Staining for ACAT1 and ABCA1 in the rheumatic mitral valve was positive beside the rheumatoid nodule (Fig. 3B-D). ACAT1 was weakly positive, but the expression of ABCA1 was not found in the control group (Fig. 3A-C).

### RT–PCR

Compared with the ischemic mitral valve, the rheumatic mitral valve presented strong expression of ABCA1, ACAT1 and the inflammatory cytokines TNF-α, IL-10, and MCP-1.

**Fig. 4:**
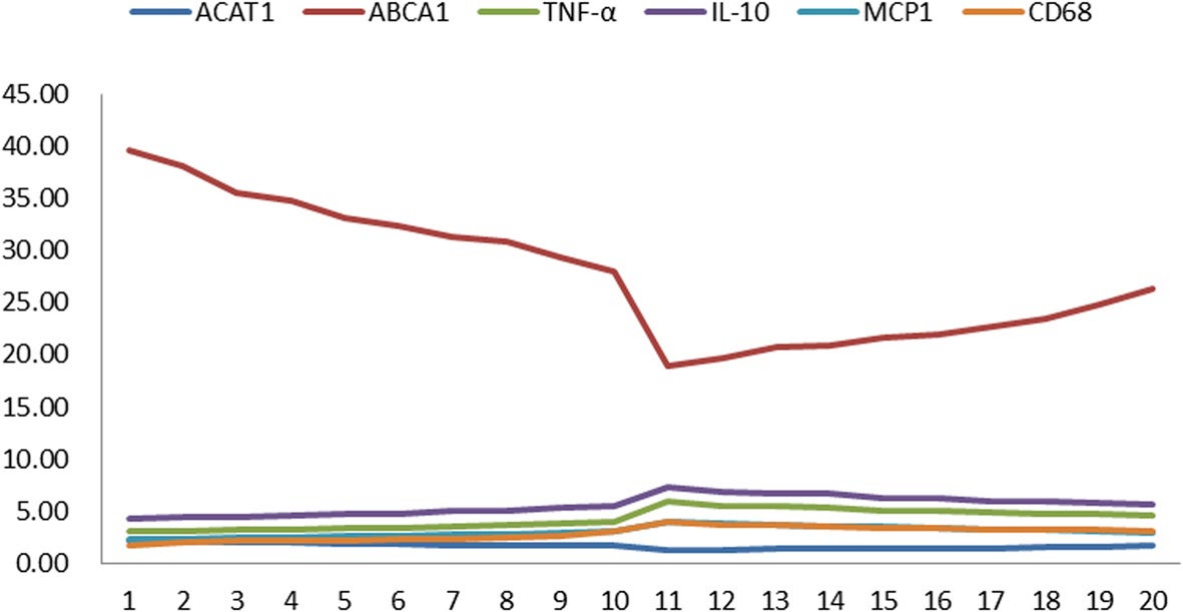
The mRNA expression levels of lipid metabolism-related genes and inflammatory factors

We further divided the rheumatic mitral valve into two groups and found that there was a significant difference between the three groups (*P* < 0.05), and a significant difference was also seen between rheumatic mitral valve-1 and rheumatic mitral valve-2 (*P* < 0.05, Table 3). In addition, the expression of ABCA1 and ACAT1 in the rheumatic mitral valve was strong but accompanied by reduced expression of inflammatory factors (Table 4, Fig. 4).

**Table 3:** The relevant mRNA expression level of lipid metabolism related genes and inflammatory factor

	*ACAT1*	*ABCA1*	*TNF-α*	*IL-10*	*MCP1*	*CD68*
*Ischemic mitral valve(n = 4)*	*1.00*	*1.00*	*1.00*	*1.00*	*1.00*	*1.00*
*Rheumatic mitral valve-1(n = 10)*	*1.86 ± 0.16*^*★*^	*33.3 ± 3.69*^*★*^	*3.41 ± 0.30*^*★*^	*4.78 ± 0.41*^*★*^	*2.60 ± 0.23*^*★*^	*2.27 ± 0.36*^*★*^
*Rheumatic mitral valve-2(n = 10)*	*1.42 ± 0.12*^*★#*^	*22.1 ± 2.30*^*★#*^	*5.11 ± 0.40*^*★#*^	*6.32 ± 053*^*★#*^	*3.45 ± 0.34*^*★#*^	*3.40 ± 0.26*^*★#*^

*ACAT1* acetoacetyl Coenzyme A thiolase − 1, *ABCA1* ATP binding cassette subfamily A member 1, *TNFα* tumour necrosis factor α, *IL10* interleukin10, *MCP* methyl cyclopentenolone

^★^there was significant difference between three groups (*P* < 0.05)

^#^there was significant difference between Rheumatic mitral valve-1and Rheumatic mitral valve-2 (*P* < 0.05)

**Table 4:** The mRNA expression level of lipid metabolism related genes and inflammatory factor

	*ACAT1*	*ABCA1*	*TNF-α*	*IL-10*	*MCP1*	*CD68*
1	2.13	39.53	3.08	4.31	2.35	1.65
2	2.07	38.06	3.10	4.34	2.36	2.06
3	1.98	35.45	3.15	4.42	2.40	2.10
4	1.94	34.73	3.21	4.50	2.45	2.14
5	1.85	33.12	3.32	4.65	2.53	2.21
6	1.81	32.40	3.39	4.75	2.59	2.26
7	1.75	31.33	3.56	4.99	2.71	2.37
8	1.72	30.79	3.63	5.09	2.77	2.42
9	1.69	29.26	3.80	5.32	2.89	2.53
10	1.68	28.04	3.91	5.47	2.98	3.02
11	1.28	18.92	5.87	7.31	4.01	3.90
12	1.31	19.70	5.51	6.81	3.87	3.66
13	1.33	20.70	5.43	6.71	3.73	3.62
14	1.34	20.85	5.25	6.64	3.55	3.50
15	1.39	21.63	5.07	6.28	3.49	3.38
16	1.41	21.94	5.00	6.19	3.38	3.33
17	1.46	22.72	4.82	5.96	3.26	3.21
18	1.51	23.50	4.79	5.87	3.23	3.18
19	1.53	24.81	4.79	5.78	3.11	3.18
20	1.65	26.37	4.61	5.66	2.92	3.06

*ACAT1* acetoacetyl Coenzyme A thiolase −1, *ABCA1* ATP binding cassette subfamily A member 1, *TNFα* tumour necrosis factor α, *IL10* interleukin10, *MCP* methyl cyclopentenolone

## Discussion

Rheumatic heart valve disease (RHVD) is an active inflammatory process prone to fibrosis and calcification [8]. Retrospective studies have shown that statin therapy is beneficial for hyperlipidemia in rheumatism. However, instead of focusing on RHVD, many studies have focused on aortic valve calcification [9, 10]. The present study addresses rheumatism and pathological mechanisms associated with lipid metabolism in an attempt to offer new insight into lipid-lowering treatment in rheumatic diseases and to provide a theoretical basis for medicine treatment and enlightenment for clinical practice.

Mitral stenosis of the left ventricle is one of the characteristic features of RHVD, and its pathogenesis is mainly considered a cross-reaction of antibodies to the Streptococcus protein M and α-helical coil protein of the myocardium. The binding of protein M to collagen type IV CB3 could lead to pathological valve damage. Pathological findings indicate that the primary sites of rheumatic damage are chronic inflammation-related injury of the perivascular endothelium under the connective tissue matrix [11]. It is generally known that in the development of atherosclerosis, endothelial cells are destroyed after inflammatory cell invasion. Lipid accumulation in blood vessels participates in the development of atherosclerosis. Based on this, it is logical to expect that lipid-blood contact valves may also cause lipid accumulation after injury to the endothelial lining of the valve. On gross observation, postoperative rheumatic heart valve specimens showed yellowish discoloration. Further histological exploration showed adipocytes, which supports our hypothesis. We also discovered, for the first time, the presence of adipose tissue in the rheumatic heart valve. Adipose tissues can not only store energy but also regulate immunity and inflammation. The presence of adipose tissues may indicate the regulation of rheumatic inflammation. However, it is uncertain how adipocytes accumulate in the rheumatic valve, and further evaluation is thereby needed in animal and human models. Previous studies reported that fibroblasts and vascular stromal cells can be converted into adipocytes [12, 13]. Since we observed fibroblasts in normal mitral valve samples, along with neovascularization, the consequences of adipose cells being converted from valve fibroblasts and vascular stromal cells during the regulation of inflammation is an obvious possibility.

After Dr. Carrey Coombs wrote the first textbook on systematic rheumatic heart disease in 1924, several scholars carried out research on rheumatic heart disease to identify the main physical changes of rheumatism as AsCHOF body, nonspecific edema and inflammatory cell infiltration [14, 15]. Molecular biology mainly focuses on the inflammatory immune mechanism caused by Streptococcus. However, the underlying mechanism of fat metabolism in rheumatic heart valve development is largely unknown. According to previous studies, hypercholesterolemia is associated with RHVD, and the use of cholesterol-lowering therapy exerts beneficial outcomes for patients [9]. Since cells develop complex mechanisms that strictly control their quantity and distribution of cholesterol, excessive free cholesterol can destroy cell membranes and produce cytotoxicity, causing blood vessels to produce plaques and calcification. Considering the homeostatic state of normal intracellular cholesterol, after cholesterol enters cells, the mechanism of cells promotes the reversal of cholesterol transport by synthesizing and increasing cholesterol. Macrophages absorb cholesterol around the tissue and convert it into bile to promote intracellular dynamic balance. In the reverse process of cholesterol transport, the most important step is efflux of cholesterol from cells. ABCA1 has been identified as a key protein in this process, and it transports free intracellular cholesterol to apolipoprotein outside the cell and reduces cholesterol content in cells. If these compensatory mechanisms fail to reduce the level of free adipocytes, ACAT1 esterifies excessive free cholesterol and adipocytes [5, 16–18]. ABCA1 was not expressed in normal valves, while ACAT1 was expressed, and the rheumatic heart valve expressed both ABCA1 and ACAT1, suggesting that lipid metabolism may recover the rheumatic valve. Previous studies indicated that lipid metabolism played an important role in the presence of the rheumatic valve. Our study is the first report, to our knowledge, of the expression of proteins associated with lipid metabolism.

Monocytes and macrophages are well-known components of the innate immune system that can regulate inflammation, tissue homeostasis, wound repair, and disease [19]. More recently, a study found that increased inflammatory cells composed of macrophages have been described in human valve diseases. At a week after birth, increasing numbers of macrophages are present, consistent with infiltrating populations of monocytes, and macrophages are localized in regions of biomechanical stress in the valve leaflets. We found a large number of macrophages in rheumatic valves, suggesting that macrophages exert a significant role in the development of rheumatic diseases. Macrophages can be briefly divided into proinflammatory macrophages and anti-inflammatory macrophages. Proinflammatory cells secrete a large number of inflammatory factors, such as TNF-a, IL-1β and IL-6, which repair damaged tissues via anti-inflammatory macrophages and promote remodeling. These cytokines may lead to progressive fibrosis of valvular lesions. Based on our results, proinflammatory factors in rheumatic heart valves were significantly higher than those in normal valves. As increased secretion of pro-inflammatory cytokines results from chronic inflammatory cells such as macrophages, it may also be from adipocytes. Previous studies have shown that adipose tissues can not only store energy but also secrete adipokines and inflammatory factors [20–22]. Adipokines are involved in rheumatic diseases such as rheumatoid arthritis and systemic lupus erythematosus [4, 23].

### Study strengths and limitation

This study provides a new insight into the potential mechanism of RHVD and lays a foundation for a new treatment scheme for RHVD in the future. However, there were several limitations that should be acknowledged in the current study. Insufficient research into the mechanism of our hypothesis exists; hence, additional in vivo and in vitro studies are warranted to confirm the current findings. Normal valvula bicuspidalis were difficult to collect, but the samples were added as a normal control group.

## Conclusions

Taken together, the present study indeed depicted that adipocytes and lipid metabolism might be involved in rheumatic heart disease. Our study is the first to discover lipid metabolism-related proteins in RHVD, which provides a new insight into the pathological mechanism of rheumatic heart disease.

## Data Availability

The datasets generated and analyzed during the current study are available from the corresponding author on reasonable request.

## Abbreviations

ABCA1: ATP-binding cassette transporter A1
ARD: Autoimmune rheumatic disease
ACAT1: Acyl-coenzyme A: cholesterol acyltransferase-1
HE: Hematoxylin–eosin
IHC: Immunohistochemical
RHVD: Rheumatic heart valve disease
RT–PCR: Real-time polymerase chain reaction
